# Targeting the stress oncoprotein LEDGF/p75 to sensitize chemoresistant prostate cancer cells to taxanes

**DOI:** 10.18632/oncotarget.15323

**Published:** 2017-02-14

**Authors:** Leslimar Ríos-Colón, Christina K. Cajigas-Du Ross, Anamika Basu, Catherine Elix, Ivana Alicea-Polanco, Tino W. Sanchez, Vinodh Radhakrishnan, Chien-Shing Chen, Carlos A. Casiano

**Affiliations:** ^1^ Center for Health Disparities and Molecular Medicine, Department of Basic Sciences, Loma Linda University School of Medicine, Loma Linda, CA 92354, USA; ^2^ Department of Medicine, Division of Hematology/Medical Oncology, Loma Linda University School of Medicine, Loma Linda, CA 92354, USA; ^3^ Department of Medicine, Division of Rheumatology, Loma Linda University School of Medicine, Loma Linda, CA 92354, USA

**Keywords:** chemoresistance, LEDGF/p75, prostate cancer, cell death, taxanes

## Abstract

Prostate cancer (PCa) is associated with chronic prostate inflammation resulting in activation of stress and pro-survival pathways that contribute to disease progression and chemoresistance. The stress oncoprotein lens epithelium-derived growth factor p75 (LEDGF/p75), also known as DFS70 autoantigen, promotes cellular survival against environmental stressors, including oxidative stress, radiation, and cytotoxic drugs. Furthermore, LEDGF/p75 overexpression in PCa and other cancers has been associated with features of tumor aggressiveness, including resistance to cell death and chemotherapy. We report here that the endogenous levels of LEDGF/p75 are upregulated in metastatic castration resistant prostate cancer (mCRPC) cells selected for resistance to the taxane drug docetaxel (DTX). These cells also showed resistance to the taxanes cabazitaxel (CBZ) and paclitaxel (PTX), but not to the classical inducer of apoptosis TRAIL. Silencing LEDGF/p75 effectively sensitized taxane-resistant PC3 and DU145 cells to DTX and CBZ, as evidenced by a significant decrease in their clonogenic potential. While TRAIL induced apoptotic blebbing, caspase-3 processing, and apoptotic LEDGF/p75 cleavage, which leads to its inactivation, in both taxane-resistant and -sensitive PC3 and DU145 cells, treatment with DTX and CBZ failed to robustly induce these signature apoptotic events. These observations suggested that taxanes induce both caspase-dependent and -independent cell death in mCRPC cells, and that maintaining the structural integrity of LEDGF/p75 is critical for its role in promoting taxane-resistance. Our results further establish LEDGF/p75 as a stress oncoprotein that plays an important role in taxane-resistance in mCRPC cells, possibly by antagonizing drug-induced caspase-independent cell death.

## INTRODUCTION

Prostate cancer (PCa) represents a significant health burden in the United States since it is the most frequently diagnosed cancer in men and the second leading cause of male cancer deaths after lung cancer (1). The rates of PCa incidence and mortality are variable among different racial groups, with African American men presenting a disproportionately high incidence and mortality compared to other ethnic/racial groups [1, 2]. Chronic inflammation of the prostate leading to an augmented state of cellular oxidative stress and activation of stress survival pathways has been linked to PCa pathogenesis and resistance to therapy [3–7].

Lens Epithelium-Derived Growth Factor of 75kD (LEDGF/p75) has recently emerged as a stress oncoprotein that promotes cellular survival against many different environmental stressors, including oxidative stress, radiation, heat, serum starvation, and cytotoxic drugs [8–20]. Also known as PC4 and SFRS1 interacting protein (PSIP1), and dense fine speckled autoantigen of 70 kD (DFS70), this protein has attracted considerable attention due to its broad relevance to cancer, autoimmunity, eye diseases, and HIV-AIDS [14, 15]. LEDGF/p75 is the target of autoantibody responses in a subset of patients with PCa [14, 21], as well as in patients with diverse chronic inflammatory conditions and some apparently healthy individuals [14]. While early studies suggested that LEDGF/p75 was a growth factor critical for the proliferation of lens epithelial cells [8], subsequent studies have demonstrated that this protein is not a lens specific growth factor but rather a ubiquitous nuclear transcription co-activator with oncogenic functions that is activated during the cellular response to stress [14, 15].

Our group and others have shown that LEDGF/p75 is upregulated in PCa and in other human cancer types, and that overexpression of this protein in cancer cells is associated with features of tumor aggressiveness, such as increased proliferation, resistance to cell death and therapy, invasion, migration, clonogenicity, angiogenesis, and tumor growth [11, 15–25]. In a previous study we reported that LEDGF/p75 overexpression in PCa cells promoted resistance against caspase-independent cell death induced through lysosomal membrane permeabilization (LMP) by the taxane drug docetaxel (DTX), the gold standard for advanced PCa chemotherapy [18]. These results were consistent with studies in other cancer cell types demonstrating that LEDGF/p75 overexpression promoted cellular protection against LMP-inducing drugs [19]. More recently, we provided evidence that LEDGF/p75 overexpression in PCa cells promotes protection against necrotic cell death induced by oxidative stress [20].

The mechanisms by which LEDGF/p75 promotes resistance to stress-induced cell death have not been fully elucidated, although available evidence suggests that this oncoprotein is upregulated or activated in response to environmental stressors [8–14, 17–20, 22, 24–25]. Acting as a transcription co-activator, it contributes to the transactivation of stress, antioxidant, and cancer-associated genes through interaction with transcription complexes involving RNA polymerase II, PC4 transcription factor, menin-MLL (mixed leukemia lineage), the MeCP2 transcription activator/repressor, and c-MYC-associated protein JPO2 [26–31]. LEDGF/p75 target genes include heat shock protein 27 (HSP27), oxidoreductase ERP57/PDIA3/GRP58, cytoglobin (CYGB), peroxiredoxin 6 (PRDX6), involucrin, alcohol dehydrogenase, aldehyde dehydrogenase, αB-crystallin, gamma glutamylcysteine synthase, vascular endothelial growth factor C (VEGF-C), and interleukin 6 (IL-6) [12, 13, 20, 22, 23, 28, 32–41].

Recent evidence points to LEDGF/p75 as a promising druggable target for HIV and leukemia therapy [42–44]. In light of our previous demonstration that LEDGF/p75 overexpression in PCa cells promoted resistance to DTX [18], the present study was conducted to determine if targeting LEDGF/p75 in chemoresistant PCa cells would re-sensitize these cells to the clinically relevant taxane drugs DTX and cabazitaxel (CBZ), which are the first and second line cytotoxic chemotherapeutic drugs, respectively, approved by the Food and Drug Administration (FDA) for the treatment of metastatic castration-resistant prostate cancer (mCRPC) [45, 46]. In addition, since LEDGF/p75 promoted protection against DTX-induced lysosomal cell death and stress-induced caspase-independent cell death in PCa cells [18, 20], we explored if the protective functions of LEDGF/p75 are linked to the ability of DTX and CBZ to activate caspase-independent mechanisms of cell death in drug-resistant PCa pre-clinical models. This study represents the first step in the development of a multi-targeting approach involving LEDGF/p75 in combination with taxanes to re-sensitize chemoresistant mCRPC cells to therapy.

## RESULTS

### LEDGF/p75 is overexpressed in DTX-resistant DU145 and PC3 cells

**Figure 1:**
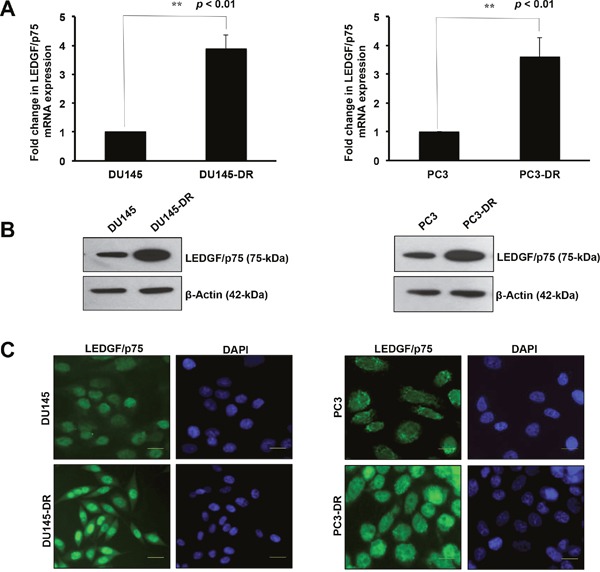
LEDGF/p75 is overexpressed in DTX-resistant DU145 and PC3 cells LEDGF/p75 transcript levels were quantified using mRNA isolated from DU145 and DU145-DR cells by qPCR in at least three independent experiments (**A**., left panel). Statistical significance was determined in comparison to control DU145 cells using Student's t-test (** p<0.01). LEDGF/p75 protein expression was evaluated in lysates from DU145 and DU145-DR cells by immunoblotting using a rabbit anti-LEDGF/p75 antibody that specifically detects this protein at 75kDa (**B**., left panel). β-actin was used as loading control. Fluorescence intensity of nuclear dense fine speckles characteristic of LEDGF/p75 was evaluated by indirect immunofluorescence microscopy in DU145 and DU145-DR cells, using a human anti-LEDGF/p75 autoantibody (**C**., left panel). LEDGF/p75 transcript levels were also quantified using mRNA isolated from PC3 and PC3-DR cells by qPCR in at least three independent experiments (**A**, right panel). P values were determined in comparison to control PC3 cells using Student's t-test (**p<0.01). LEDGF/p75 protein expression was evaluated in lysates from PC3 and PC3-DR cells by immunoblotting using a rabbit anti-LEDGF/p75 antibody (**B**, right panel). β-actin was used as loading control. Fluorescence intensity of nuclear dense fine speckles characteristic of LEDGF/p75 was evaluated by indirect immunofluorescence microscopy in PC3 and PC3-DR cells, using a human anti-LEDGF/p75 autoantibody (**C**, right panel).

We determined the expression of LEDGF/p75 in the DTX-resistant mCRPC cell lines DU145-DR and PC3-DR, compared to the drug sensitive parental DU145 and PC3 cells. These cells were developed to be resistant to DTX by selecting and expanding the surviving cells after successive treatments with increasing concentrations of the drug. We observed that the expression of LEDGF/p75 was significantly upregulated at the transcript and protein level as DU145 and PC3 cells made the transition from chemosensitivity to chemoresistance (Figure 1). The DU145-DR cells displayed a significant 3.87 fold increase in LEDGF/p75 transcript expression compared to the parental DU145 cells (Figure 1A, left panel). To determine if the increase in transcript expression correlated with increased protein expression, we collected total lysates from DU145 and DU145-DR cells and performed immunoblotting using an antibody specific for LEDGF/p75. We observed that LEDGF/p75 was robustly expressed in the DU145-DR cells compared to the parental cells (Figure 1B, left panel). To further confirm these findings, we then proceeded to analyze LEDGF/p75 expression by immunofluorescence microscopy using a well-characterized human autoantibody against this protein [47]. Acquiring the images under exactly the same parameters, we observed that the fluorescence intensity of nuclear dense fine speckles, corresponding to LEDGF/p75 staining [28, 47], in the DU145-DR cells was higher compared to the intensity in the chemosensitive DU145 cells (Figure 1C, left panel).

The same experimental procedures were performed to assess LEDGF/p75 expression in the PC3 and PC3-DR cells. When we compared the LEDGF/p75 transcript expression in these two cell lines, we observed a significant 3.60 fold increase in the transcript levels in PC3-DR compared to the sensitive PC3 cells (Figure 1A, right panel). As in DU145-DR cells, there was a robust increase in LEDGF/p75 protein expression in the PC3-DR cells compared to the parental PC3 cells (Figure 1B, right panel). Also, similar to DU145-DR cells, the fluorescence intensity of LEDGF/p75 staining in PC3-DR cells was higher when compared under identical imaging conditions to the PC3 cells (Figure 1C, right panel). Taken together, these findings showed higher endogenous expression of LEDGF/p75 in DTX-resistant cells at both the transcript and protein levels compared to their drug-sensitive parental cells.

### DU145-DR and PC3-DR cells are resistant to multiple taxanes but not to TRAIL

We then investigated if the DTX-resistant PCa cells, which showed endogenous overexpression of LEDGF/p75, were selectively resistant to DTX or also showed multi-drug resistance, particularly to other taxanes such as CBZ and paclitaxel/taxol (PTX). Currently, CBZ is the second-line cytotoxic chemotherapeutic drug available for advanced PCa patients that develop resistance to DTX [45, 46]. We also included in our analysis PTX, the original taxane and parent drug of both DTX and CBZ. Although not currently used for clinical treatment of advanced PCa, PTX is commonly used in the treatment of other tumor types [48], and it was therefore important to determine if DTX-resistant cancer cells overexpressing LEDGF/p75 also promote resistance to this parental taxane. We also treated our chemosensitive and chemoresistant PCa cells with tumor necrosis factor related apoptosis inducing ligand (TRAIL), an inducer of caspase-dependent apoptosis. In previous studies we observed that while ectopic LEDGF/p75 overexpression promoted protection against stressors that induced caspase-independent cell death such as DTX and tert-butyl hydroperoxide (TBHP), it did not confer protection against classical inducers of caspase-dependent cell death such as TRAIL and staurosporine (STS) [18, 20]. Therefore, we sought to reproduce these observations in chemoresistant PCa cells naturally overexpressing LEDGF/p75 after selection.

**Figure 2:**
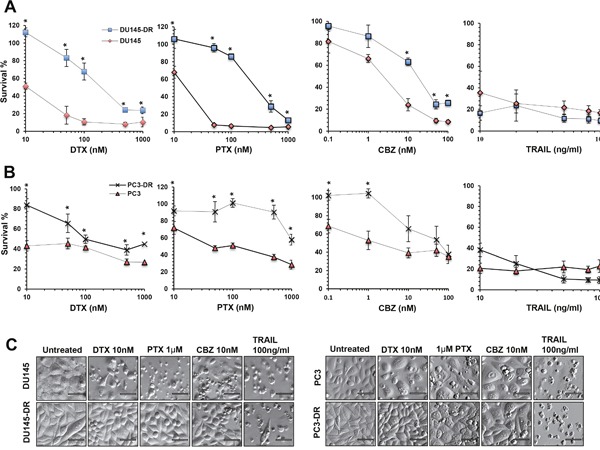
DU145-DR and PC3-DR cells are resistant to multiple taxanes but not to TRAIL **A**. and **B**. Assessment of cell viability as measured by MTT assay in DU145 (red diamonds), DU145-DR (blue squares), PC3 (red triangles), and PC3-DR (crosses) cells treated with increasing concentrations of DTX (10nM, 50nM, 100nM, 500nM, and 1000nM), PTX (10nM, 50nM, 100nM, 500nM and 1000nM), CBZ (0.1 nM, 1nM, 10nM, 50nM and 100nM) for up to 72 hr, and TRAIL (10ng/ml, 20ng/ml, 50ng/ml, 80ng/ml and 100ng/ml) for 24 hr. Each graph represents the average of at least three different experiments in triplicates normalized to untreated controls. Standard error of the mean (SEM) was calculated. Statistical significance was determined by comparing the values for each drug concentration in the DTX-sensitive DU145 and PC3 cells versus the DTX-resistant DU145-DR and PC3-DR cells, respectively, using Student's t-test (*p<0.05). **C**. Morphology of drug-sensitive and –resistant cells after treatment with DTX, PTX, CBZ, or TRAIL, visualized by Hofmann Modulation Contrast microscopy. Scale bar set at 20 μM.

For these studies, we treated DU145-DR cells and DU145 cells with increasing concentrations of DTX, PTX, or CBZ for up to 72 hr, and TRAIL for up to 24 hr (Figure 2A) and determined the approximate drugs’ half-maximal effective concentrations (EC50). We observed higher viability in DU145-DR cells treated with DTX (EC50 ≈ 200 nM), and PTX (EC50 ≈ 300 nM), compared to DU145 cells, which showed EC50s of 10 nM and 20 nM, respectively. In the dose response curves, a statistically significant difference in the percentage of surviving cells could be observed at all concentrations (range 10-1000 nM). Interestingly, when we examined the difference in the overall survival between DU145-DR and DU145 cells treated with CBZ, there was a trend toward higher survival in the DU145-DR cells, with EC50 values of 11 nM in the DU145-DR cells and 2 nM in the DU145 cells. For CBZ, there was a statistically significant difference in survival at concentrations above 10 nM. We did not observe a difference in survival when DTX-resistant and -sensitive cell lines were treated with increasing concentrations of TRAIL. Both cell lines showed high sensitivity to low concentrations of this cell death ligand (10 ng/ml).

Similar experiments with PC3-DR and PC3 cells revealed a statistically significant difference in the percentage of surviving PC3-DR cells compared to PC3 cells after exposure to DTX, with EC50s of 100 nM and 10 nM, respectively (Figure 2B). We also observed a statistically significant increased survival in PC3-DR cells exposed to PTX, with an EC50 of 1000 nM, compared to an EC50 of 100 nM for PC3 cells. Consistent with results obtained with the DU145-DR and DU145 cells, there was an overall trend in the difference in survival between PC3-DR cells and PC3 cells after treatment with CBZ. We obtained EC50s of 50 nM for PC3-DR and 5 nM for PC3 cells, with statistically significant differences at CBZ concentrations below 1 nM. Like in DU145 cells, we did not observe any differences when PC3-DR and PC3 cells were treated with TRAIL.

We then proceeded to examine the morphology of the cells under the different treatment conditions (Figure 2C). We observed that DTX-resistant cells had a relatively normal morphology with fewer floating cells and features of cell death, compared to the sensitive cell lines which clearly showed increased cell death when treated with DTX, PTX, or CBZ at the low pharmacological concentrations of 10 nM, 1 μM, and 10 nM, respectively. Robust apoptotic cell death could be observed upon treatment with TRAIL. In summary, there was increased cell survival in the DTX-resistant cell lines, which express high endogenous levels of LEDGF/p75, during treatment with increasing concentrations of the different taxanes. However, none of the cell lines showed resistance to TRAIL.

### LEDGF/p75 depletion sensitizes DTX-resistant PCa cells to clinically relevant taxanes

Given that the transition from taxane sensitivity to resistance in PCa cells involves the upregulation of several survival pathways [49, 50], it was necessary to establish the contribution of LEDGF/p75 to the observed taxane resistance in DU145-DR and PC3-DR cells. For these experiments, we transiently knocked down LEDGF/p75 in our drug-resistant models using small inhibitory RNAs (siRNAs) specific for this protein [20, 33]. We sought to determine if LEDGF/p75 knockdown alone decreased the clonogenic potential of taxane-resistant cells, and if its combination with drug treatment further sensitized the cells to taxane chemotherapy. We chose clonogenic assays for these experiments because they could clearly show cellular sensitization to the treatments over time by decrease in colony formation. The spatial constraints (96 well plates) that we encountered in short-term MTT viability assays did not permit to assess the long-term effects of drugs on cell growth. Colony formation assays have been widely used to determine the effects of LEDGF/p75 knockdown on its tumorigenic properties as well as its ability to promote resistance to non-taxane drugs in non-PCa cell models [19, 23, 25, 27, 51].

**Figure 3:**
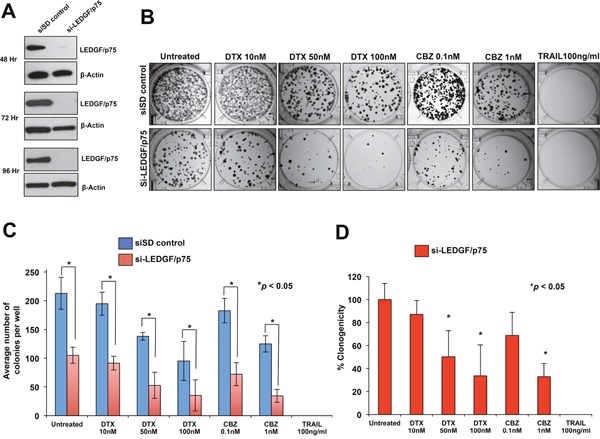
Transient knockdown of LEDGF/p75 sensitizes DU145-DR cells to clinically relevant taxanes DTX and CBZ **A**. LEDGF/p75 knockdown was confirmed by immunoblotting using a rabbit anti-LEDGF/p75 antibody in DU145-DR cells transfected with si-LEDGF/p75 oligos as compared to cells transfected with the siSD control oligos. **B**. Representative images of colony formation assay plates showing a decrease in clonogenicity in DU145-DR cells with LEDGF/p75 depletion compared to siSD control cells, in the presence and absence of drugs. Colonies were counted after 12 days of treatment. **C**. Bar graph showing quantification of DU145-DR colonies. Each bar represents the average of colonies counted in at least three independent experiments. SEM was calculated. **D**. Bar graph showing the percent clonogenicity in DU145-DR cells with LEDGF/p75 depletion, in the presence and absence of drug treatment, compared to untreated cells. Data was derived from the bar graph shown in panel C. SEM was calculated. Statistical significance was determined by comparing the values for cells transfected with siSD control oligos vs cells with LEDGF/p75 knockdown, in the presence or absence of drugs, using Student's t-test (*p<0.05).

**Figure 4:**
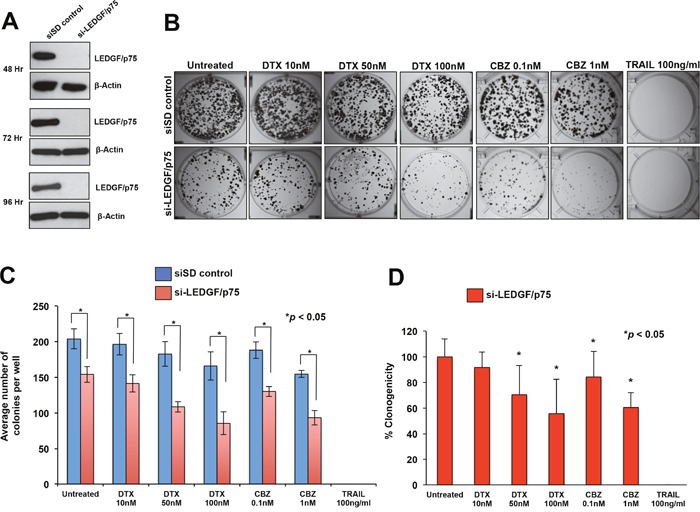
Transient knockdown of LEDGF/p75 sensitizes PC3-DR cells to clinically relevant taxanes DTX and CBZ **A**. LEDGF/p75 knockdown was confirmed by immunoblotting using a rabbit anti-LEDGF/p75 antibody in PC3-DR cells transfected with si-LEDGF/p75 oligos as compared to cells transfected with the siSD control oligos. **B**. Representative images of colony formation assay plates showing a decrease in clonogenicity in PC3-DR cells with LEDGF/p75 depletion compared to siSD control cells, in the presence and absence of drugs. Colonies were counted after 12 days of treatment. **C**. Bar graph showing quantification of PC3-DR colonies. Each bar represents the average of colonies counted in at least three independent experiments. SEM was calculated. **D**. Bar graph showing the percent clonogenicity in PC3-DR cells with LEDGF/p75 depletion, in the presence and absence of drugs, compared to untreated cells. Data was derived from the bar graph shown in panel C. SEM was calculated. Statistical significance was determined by comparing the values for cells transfected with siSD control oligos vs cells with LEDGF/p75 knockdown, in the presence or absence of drugs, using Student's t-test (*p<0.05).

Transient knockdown of LEDGF/p75 in DU145-DR and PC3-DR cells led to robust depletion of the protein compared to cells transfected with scrambled duplex siRNA (siSD) control (Figure 3A and 4A). LEDGF/p75 depletion was still robust 96 hr post-transfection in both cell lines, indicating that it was stable at the time the cells were treated with the DTX or CBZ during the initial hours of clonogenic growth (Figure 3A and 4A). We focused on DTX and CBZ on these and subsequent experiments because they are the first and second line chemotherapeutic drugs, respectively, currently used for the clinical treatment of mCRPC.

LEDGF/p75 depletion alone in DU145-DR cells in the absence of taxanes significantly reduced colony formation by 50.7% compared to untreated siSD control cells (Figure 3B, 3C, 3D). On the other hand, treatment with DTX alone, without LEDGF/p75 depletion, significantly reduced colony formation by 35% and 55.3% at concentrations of 50 nM and 100 nM, respectively, compared to the untreated siSD control cells (Figure 3C), indicating an EC50 for DTX alone of approximately 100 nM. However, the combination of LEDGF/p75 depletion plus 50 nM DTX or 100 nM DTX reduced colony formation by 75.2% and 83.4%, respectively, when compared to untreated SD-control cells (Figure 3C), and by 49.7% and 66.4%, respectively, when compared to LEDGF/p75 depletion alone (Figure 3D). These results indicated that the combination of DTX plus LEDGF/p75 depletion chemosensitized the cells, with EC50 < 50 nM, compared to untreated cells.

In the case of CBZ, this drug alone, without LEDGF/p75 depletion, significantly reduced colony formation in DU145-DR cells by 14.2% and 41.8% at concentrations of 0.1 nM and 1 nM, respectively, when compared to untreated siSD-control cells (Figure 3B, 3C), indicating an EC50 above 1 nM. However, the combination of LEDGF/p75 depletion plus 0.1 nM CBZ or 1 nM CBZ reduced colony formation by 66.1% and 83.8%, respectively, when compared to untreated siSD control cells (Figure 3C), and by 31.2% and 67.1%, when compared to LEDGF/p75 depletion alone (Figure 3D). These results indicated that the combination of CBZ plus LEDGF/p75 depletion chemosensitized the cells, with EC50 < 0.1 nM, compared to untreated cells.

DU145-DR cells transfected with siSD control oligos or si-LEDGF/p75 were equally sensitive to 100 ng/ml TRAIL, a concentration used in previous studies to efficiently induce caspase-dependent apoptosis [18, 21] and which did not yield any colonies (Figure 3B, 3C, 3D). These results were consistent with results shown in Figure 2 in which DU145 cells, both DTX sensitive resistant, were equally sensitive to a wide range of TRAIL concentrations (10 ng/ml-100 ng/ml).

We then performed similar studies to determine the effects of transient LEDGF/p75 depletion in PC3-DR cells, with and without drug treatment. We observed a significant decrease (24.4%) in the clonogenicity of PC3-DR cells after LEDGF/p75 depletion compared to the siSD control cells, although the effect was not as robust as in the DU145-DR cells (Figure 4B, 4C). Treatment with DTX alone, without LEDGF/p75 depletion, reduced PC3-DR colony formation by 10.4% and 18.6% at concentrations of 50 nM and 100 nM, respectively, compared to the untreated siSD control cells (Figure 4C), indicating an EC50 well above 100 nM. However, the combination of LEDGF/p75 depletion plus 50 nM DTX or 100 nM DTX reduced colony formation in the PC3-DR cells by 46.7% and 57.9%, respectively, when compared to untreated SD-control cells (Figure 4C), and by 29.5% and 44.4%, respectively, when compared to LEDGF/p75 depletion alone (Figure 4D). These results indicated that the combination of DTX plus LEDGF/p75 depletion chemosensitized the cells, with an EC50 between 50 and 100 nM, compared to untreated cells.

Treatment of PC3-DR cells with CBZ alone, without LEDGF/p75 depletion, significantly reduced clonogenicity by 7.7% and 24.1% at concentrations of 0.1 nM and 1 nM, respectively, when compared to untreated siSD-control cells (Figure 4C), indicating an EC50 well above 1 nM. However, the combination of LEDGF/p75 depletion plus 0.1 nM CBZ or 1 nM CBZ reduced PC3-DR colony formation by 36.2% and 54.2%, respectively, when compared to untreated siSD control cells (Figure 3B), and by 15.6% and 39.4%, when compared to LEDGF/p75 depletion alone (Figure 4D). These results indicated that the combination of CBZ plus LEDGF/p75 depletion chemosensitized the cells, with EC50 < 1 nM, compared to untreated cells.

Like in DU145-DR cells, we also observed that PC3-DR cells transfected with siSD control oligos or si-LEDGF/p75 were equally sensitive to 100 ng/ml TRAIL. Taken together, these results showed that LEDGF/p75 depletion in DU145-DR and PC3-DR cells significantly diminished their clonogenicity, an effect that was enhanced in combination with taxanes.

### LEDGF/p75 retains structural integrity during taxane-induced cell death but is robustly cleaved during TRAIL-induced apoptosis

We showed previously that PCa cells with ectopic overexpression of LEDGF/p75 were more resistant to DTX-induced lysosomal cell death and to oxidative stress-induced necrosis, but not to classical apoptosis inducers such as TRAIL and stauroporine (STS) [18, 20]. In addition, studies from our group showed that during caspase-dependent cell death triggered by classical apoptosis inducers (e.g., Fas, TRAIL, STS, etoposide), LEDGF/p75 is cleaved by caspases-3 and -7, generating various cleavage fragments, including a signature fragment of 65 kD that lacks pro-survival activity and exacerbates cell death in the presence of stress [11, 20–21]. We also demonstrated that this protein has a short splice variant, LEDGF/p52, which induces apoptosis when ectopically overexpressed leading to LEDGF/p75 cleavage and impaired ability to transactivate stress survival genes [52]. Together, these observations suggested that the stress protective effects of LEDGF/p75 are more relevant in the context of cellular resistance to stress-induced caspase-independent cell death, where lack of caspase activation results in preservation of LEDGF/p75 structural integrity, which is essential for its transcriptional and stress survival functions [11, 32, 52].

**Figure 5:**
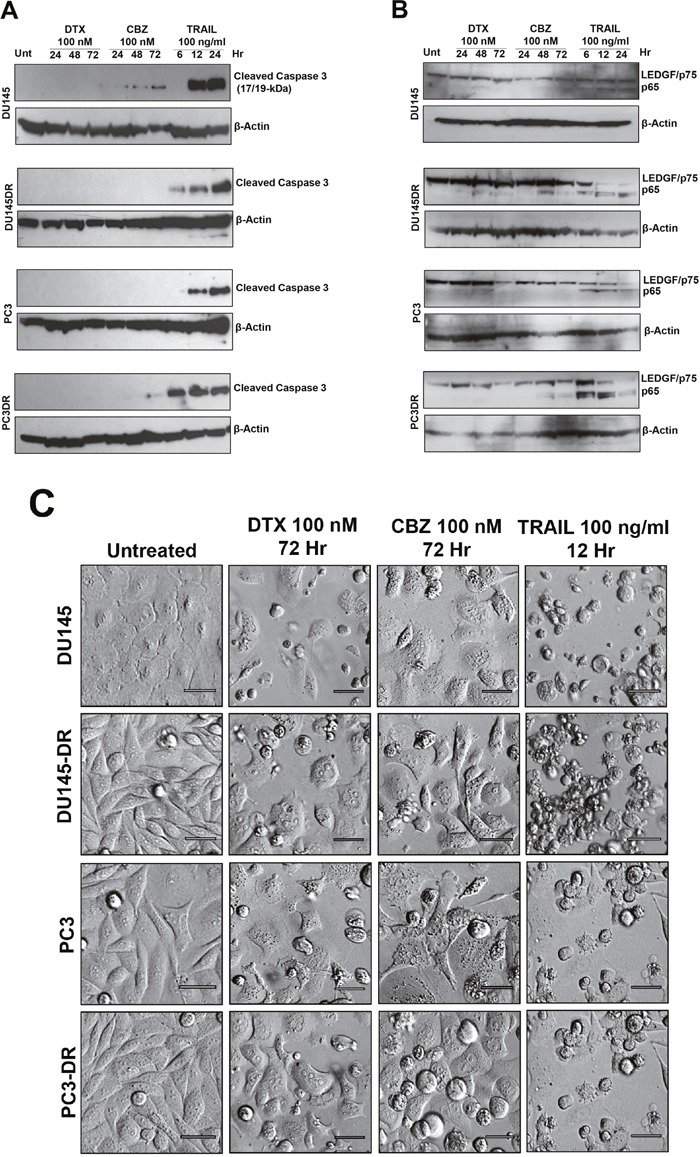
Caspase-3 processing and LEDGF/p75 cleavage in mCRPC cells treated with taxanes and TRAIL **A**. Caspase-3 processing was detected by immunoblotting using an antibody specific for its large subunit (17/19 kD). Whole lysates were obtained from DU145, DU145-DR, PC3 and PC3-DR cells after treatment with DTX or CBZ (100nM) for 24, 48 and 72 hr, or TRAIL (100ng/ml) for 6, 12, and 24 hr. Untreated cells were used as controls. β-actin was used as loading control. **B**. LEDGF/p75 cleavage was assessed using a human anti-LEDGF/p75 autoantibody that specifically detects this protein (75 kD) and its main apoptotic cleavage fragment (65 kD). Whole lysates were obtained from cells after treatment with DTX or CBZ (100nM) for 24, 48 and 72 hr, or TRAIL (100ng/ml) for 6, 12, and 24 hr. Untreated cells were used as controls. β-actin was used as loading control. **C**. Cell morphology was assessed by Hofmann Modulation Contrast microscopy to visualize features of cell death after drug treatment. Scale bar set at 20 μM.

In light of these previous observations and the observed contribution of LEDGF/p75 to cellular resistance to taxanes but not TRAIL, we designed experiments to determine if the structural integrity of LEDGF/p75 is preserved during taxane-induced cell death. For these studies, we treated DTX-sensitive and -resistant DU145 and PC3 cells with concentrations of DTX (100 nM), CBZ (100 nM), or TRAIL (100 ng/ml) that caused cell death in the previous experiments (Figures 2-4). We first examined by Western blotting if there was caspase-3 processing under these treatments, which would be indicative of activation of caspase-dependent apoptosis, using an antibody that specifically recognizes cleaved caspase-3 (Figure 5A). We observed the appearance of strong bands corresponding to cleaved caspase-3 fragment in lysates from both DTX-sensitive and -resistant cells treated with TRAIL. However, we did not detect robust processing of caspase-3 in DU145 or PC3 cells, both sensitive and resistant, 72 hr after exposure to 100 nM DTX, in spite of observing significant cell death at this concentration and time point, particularly in the sensitive cells, in previous experiments (Figure 2). Overexposure of chemiluminescent blots to film did not result in increased detection of caspase-3 cleavage in cells treated with DTX or CBZ (data not shown). There was a slight detection of cleaved caspase-3 in DU145 cells treated with 100 nM CBZ (Figure 5A), in spite of the extensive loss of cell survival observed at this concentration in previous experiments (Figure 2). These experiments suggested that while TRAIL clearly induces caspase-dependent cell death in these cell lines, both sensitive and resistant, DTX and CBZ did not induce a comparatively robust caspase-3 processing and activation at the relatively high pharmacological concentrations used.

We then proceeded to determine if LEDGF/p75 was cleaved during the same experimental conditions. As expected, we observed robust cleavage of LEDGF/p75 into its signature apoptotic 65 kD fragment in DU145 and PC3 cells, both sensitive and resistant, treated with TRAIL (Figure 5B). This fragment was detected using human anti-LEDGF/p75 autoantibodies, which recognize a C-terminal autoepitope region that is preserved in the apoptotic cleavage fragments [11, 20]. However, LEDGF/p75 was minimally cleaved during the 72 hr treatment with 100 nM DTX in all cell lines (Figure 5B). Although cleavage was more visible, albeit still weak, in cells treated with 100 nM CBZ, it did not achieve the robustness of the cleavage induced by TRAIL (Figure 5B). These results were consistent with the observed minimal processing of caspase-3 during taxane-induced cell death, with robust processing during TRAIL-induced apoptosis (Figure 5A).

In light of these results we proceeded to examine closely the morphology of DU145 and PC3 cells, both DTX-sensitive and -resistant, treated with 100 nM DTX, 100 nM CBZ, or 100 ng/ml TRAIL to assess the features of cell death (Figure 5C). The TRAIL-treated cells exhibited the classical apoptotic morphology characterized by extensive blebbing and shrinkage. By contrast, cells exposed to DTX and CBZ exhibited rounding and floating, consistent with mitotic arrest and catastrophe, as well as cells that appeared to be swollen and with breakage of the cell membrane. While there were cells displaying apoptotic blebbing, this feature was not as prominent in the taxane-treated cells as in the cells treated with TRAIL. These results are consistent with the observation of robust caspase-3 processing and LEDGF/p75 cleavage during TRAIL-induced cell death (Figure 5A) but not during taxane treatment (Figure 5B).

### LEDGF/p75 depletion does not influence the expression of the multidrug resistance protein P-glycoprotein in DTX-resistant PCa cells

**Figure 6:**
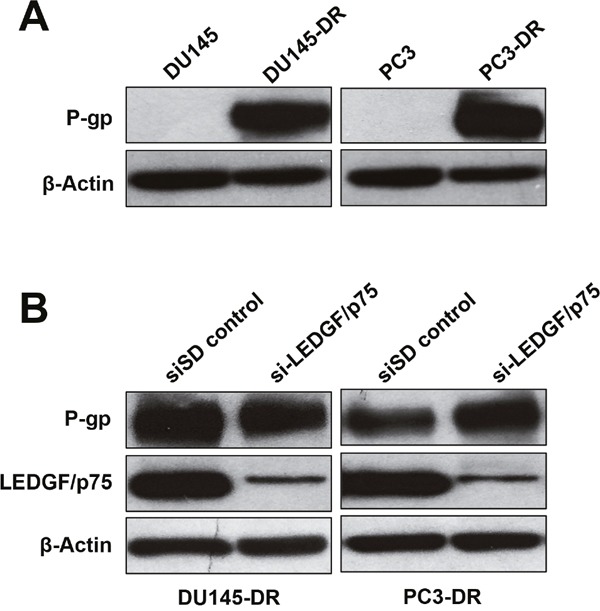
LEDGF/p75 depletion in DTX-resistant cells does not lead to downregulation of P-glycoprotein **A**. Immunoblots showing increased P-glycoprotein (P-gp) expression in DU145-DR and PC3-DR cells compared to the DTX sensitive, parental cell lines. **B**. Immunoblots showing that transient depletion (72 hr) of LEDGF/p75 in DU145-DR and PC3-DR does not attenuate P-gp expression. β-actin was used as loading control.

The molecular mechanisms by which LEDGF/p75 promotes taxane resistance are relatively unknown, although they are likely linked to its ability to transcriptionally co-activate stress survival genes. Given the established role of multidrug resistance or transporter proteins such as P-glycoprotein (P-gp, also known as ABCB1 or MDR1) in PCa chemoresistance [53, 54], we speculated that LEDGF/p75 might upregulate this protein in taxane resistant cells. For these experiments, we first compared the expression of P-gp in DU145-DR and PC3-DR cells to the drug-sensitive, parental DU145 and PC3 cells. Consistent with its role in chemoresistance, P-gp was not expressed in the sensitive cell lines but was highly expressed in the drug-resistant cells (Figure 6A). We then determined if transient LEDGF/p75 depletion (up to 72h) in the taxane resistant cells led to downregulation of P-gp. Our results indicated that LEDGF/p75 depletion had no effect on P-gp expression levels in DU145-DR and PC3-DR, suggesting that P-gp is not a target gene of this stress transcription co-activator.

## DISCUSSION

Our understanding of mechanisms underlying mCRPC has improved the outcomes for the management of this disease, with new therapeutic regimens that include sipuleucel-T, denosumab, abiraterone acetate, enzalutamide, and taxane therapy [55]. Unfortunately, despite these advances and overall increase in patient survival, mCRPC is still a challenging disease to manage, with most patients dying within three years of diagnosis due to therapy resistance, particularly to taxanes [49, 50, 53, 55–58]. The goal of the present study was to further establish the role of LEDGF/p75 in PCa taxane resistance and its potential as a novel therapeutic target for overcoming this resistance. Our group and others have provided evidence for the role of this protein in promoting tumor aggressive properties and chemoresistance in various cancer types [11, 17–20, 22–27]. We reported previously that stable overexpression of LEDGF/p75 in PC3 cells attenuated DTX-induced caspase-independent cell death caused by LMP, as well as oxidative stress-induced necrosis, but not to the apoptosis inducers TRAIL and STS [18, 20]. Given its role as a stress transcription coactivator, LEDGF/p75 activation by cancer cells is likely to counter rapid increases in oxidative stress that might overwhelm cellular antioxidant defense mechanisms and induce DNA damage and LMP.

In this study we used mCRPC cellular models (PC3 and DU145) that were selected over time for their acquired resistance to DTX. While multiple mechanisms of taxane-resistance likely operate in these cells, we focused on LEDGF/p75 given our previous observations linking its ectopic overexpression to DTX resistance in mCRPC cells [18]. Our results clearly demonstrated the upregulation of endogenous LEDGF/p75 in DU145-DR and PC3-DR cells. This upregulation did not appear to occur during short-term exposure to DTX because cells treated with increasing concentrations of DTX for up to 48 hr did not show LEDGF/p75 upregulation (data not shown). However, as we selected chemoresistant clones after weeks of exposure to increasing concentrations of DTX, we began to detect elevated endogenous LEDGF/p75 levels, suggesting that this stress protein contributes to the selection of surviving cells in the presence of DTX.

Our results also showed that DTX-resistant DU145 and PC3 cells displayed increased resistance to PTX and CBZ, compared to sensitive cells, at a wide range of pharmacological concentrations. However, both DTX-sensitive and -resistant cell lines showed robust sensitivity to TRAIL. These results suggested that the mechanisms of DTX resistance operating in these cells could also apply to PTX and CTX resistance, but not to TRAIL. The exquisite sensitivity of taxane-resistant cells to TRAIL suggests that apoptosis induction via death receptor signaling could be used to bypass the cellular protective functions of LEDGF/p75 and other survival proteins that are susceptible to caspase-mediated cleavage and inactivation. Unfortunately, efforts to target TRAIL receptors in clinical trials have been tempered by increased toxicity to cancer patients, most likely due to the high levels of these receptors in normal tissues [59].

Recent efforts to target LEDGF/p75 in the context of HIV-AIDS and leukemia have provided “proof-of-principle” that this protein is a druggable molecular target [27, 43, 44, 51, 60]. We reasoned that if LEDGF/p75 upregulation occurs during development of taxane chemoresistance, then targeting this protein in pre-clinical mCRPC models in combination with taxanes would sensitize sub-populations of resistant tumor cells to these drugs. Our results revealed that transient LEDGF/p75 depletion by itself, without drug treatment, attenuated the clonogenicity of both DTX-sensitive and -resistant PC3 and DU145 cells, consistent with results from other groups using other tumor cell models [19, 25]. It should be noted, however, that LEDGF/p75 is not essential for cell viability under normal growth conditions since cancer cell lines with stable knockdown of this protein have been effectively developed [20, 28]. Also, PSIP1/LEDGF/p75^−\−^ knockout mice were viable despite suffering from multiple skeletal malformations leading to increased perinatal mortality [61]. However, under stress conditions, LEDGF/p75 plays a key role in enhancing cell survival [13, 14].

Our results also showed that LEDGF/p75 depletion in combination with DTX or CBZ significantly decreased the clonogenic potential of both DU145-DR and PC3-DR cells, particularly at higher, albeit still pharmacological, drug concentrations. Given the limited range of taxane concentrations used in our clonogenic assays, it was not possible to determine with precision the exact EC50 values for drug-resistant DU145 and PC3 cells treated with either DTX or CBZ, with or without LEDGF/p75 depletion. While our results suggest a possible additive effect of the combinatorial treatment of taxanes plus LEDGF/p75 depletion, we cannot rule out the possibility that this combination acts synergistically to re-sensitize resistant cells. This could be explored in future studies by applying the Chou Talalay statistical method [62] to results from experiments in which a broad range of concentrations of both taxanes and small molecule inhibitors of LEDGF/p75 are combined. Our data also indicated that the anti-survival effects of LEDGF/p75 silencing was more pronounced in DU145-DR cells, consistent with the previous observation that LEDGF/p75 silencing in DU145 cells impairs their aggressive properties [23].

LEDGF/p75 silencing did not completely sensitize resistant cells to DTX and CBZ, most likely due to the contribution of other independent mechanisms, possibly involving clusterin and P-gp drug transporter, to taxane resistance [49, 50, 53, 54]. Indeed, our results demonstrated that LEDGF/p75 depletion does not downregulate P-gp in taxane-resistant cells, suggesting that these two proteins act independently of each other. We cannot rule out, however, that LEDGF/p75 may transcriptionally activate P-gp in resistant cells but the cellular stability of this drug transporter may not make it susceptible to downregulation in response to LEDGF/p75 depletion. Interestingly, a previous study showed that while P-gp is dramatically upregulated in several chemoresistant PCa cell lines, its inhibition reversed the resistant phenotype on a cell-line dependent manner, which would be consistent with the notion that multiple mechanisms of drug resistance may be activated in prostate tumor cells in response to chemotherapy [54]. Identifying resistance mechanisms independent of P-gp is therefore important since targeting this drug-transporter has not been highly successful because of the complexity of toxicity, adverse side effects, and altered pharmacokinetics encountered in studies [63].

Maintaining the structural integrity of LEDGF/p75, particularly its C-terminal domain, is essential for its transcriptional and stress survival functions [11, 32, 52]. During apoptosis, caspase-3 mediated cleavage removes the extreme N-terminal and C-terminal regions of LEDGF/p75, abrogating its stress survival functions [11]. This could explain why LEDGF/p75 overexpression in cancer cells typically does not confer protection against insults that robustly activate caspase-3, such as TRAIL and STS, which result in LEDGF/p75 cleavage and inactivation, but does protect against insults that induce LMP and even necrotic cell death, which leave the protein relatively intact [18, 19, 64]. While several reports have underscored the anti-apoptotic role of LEDGF/p75 in cancer cells, the exact mode of cell death, and the possibility that caspase-dependent and caspase-independent pathways operate in parallel under the experimental conditions used, have not been fully characterized in these studies [17, 23, 24, 65]. Nevertheless, it is plausible that LEDGF/p75 could promote protection against apoptosis if this mode of cell death occurs downstream of events antagonized by this protein such as DNA damage and LMP [18–19, 24, 64].

**Figure 7:**
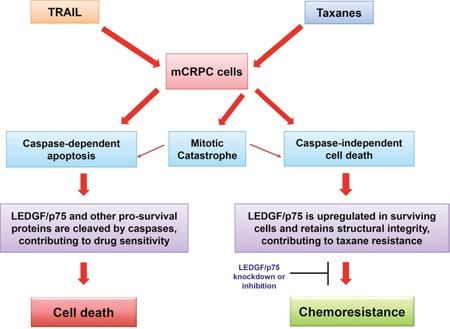
Model illustrating the potential role of LEDGF/p75 in the attenuation of drug-induced caspase-dependent and –independent cell death

Our observation that DTX and CBZ did not induce robust caspase-3 processing and LEDGF/p75 cleavage at relatively high pharmacological plasma concentrations that induce cell death (100 nM), suggests that insufficient apoptosis induced by taxanes may lead to drug resistance by preserving the structural integrity of LEDGF/p75 and other stress survival proteins that otherwise would be cleaved and inactivated during apoptotic caspase-3 activation (Figure 7). In a previous study, we showed that caspase-3 activity and LEDGF/p75 cleavage could be induced in PC3 cells, albeit not robustly, at micromolar concentrations of DTX [18]. While high micromolar DTX concentrations are detected in plasma of cancer patients early after drug administration, typically they drop to the low nanomolar range a few days post-treatment, which may prevent induction of robust and sustained tumor cell apoptosis [66]. Consistent with this, a recent study indicated that intratumoral accumulation of DTX and CBZ is key for their efficacy, with development of DTX resistance occurring if accumulation of this drug is insufficient [67].

The limited caspase-3 processing and LEDGF/p75 cleavage in taxane treated mCRPC cells observed in this study is consistent with the recent observation that DTX is a poor inducer of caspase-dependent apoptosis in DU145 cells [68], and our previous observation that DTX induces both caspase-dependent and caspase-independent lysosomal cell death in PC3 cells [18]. It is likely that induction of both caspase-dependent and -independent cell death by taxanes occurs in parallel in the tumor microenvironment, and that depending on the cellular context and local drug concentration, one cell death mode may be preferred over the other [18, 69, 70]. This then raises the intriguing question of whether there are intrinsic mechanisms in mCRPC tumors, such as upregulation of LEDGF/p75 and other stress oncoproteins, that favor promoting resistance to taxane-induced caspase-independent cell death. Although the mechanisms of cellular resistance to taxanes are not completely understood, current evidence points to tubulin mutations, multidrug transporters, androgen receptor-mutations, and overexpression of transcription factors and stress proteins such as Stat1, Stat3, NF-kB, Hsp27, and Clusterin [49, 50, 53, 54, 71]. It remains to be established, however, whether some of these mechanisms effectively antagonize taxane-induced caspase-independent cell death. If this turns out to be the case, then targeting multiple molecular pathways that protect tumor cells against both caspase-dependent and caspase-independent cell death could be an effective strategy to overcome taxane resistance in mCRPC. Since LEDGF/p75 appears to be a novel promoter of mCRPC cell resistance to taxane-induced caspase-independent cell death, this protein could be considered as a promising therapeutic target to overcome this resistance in combinatorial therapies.

Finally, understanding the complex mechanisms underlying LEDGF/p75-promoted taxane resistance will require a close examination of the regulatory mechanisms controlling the expression of this stress protein during PCa transition to chemoresistance. To date, known mechanisms of LEDGF/p75 regulation include its activation by transcription factor Sp1 [72, 73], as well as repression by sumoylation, TGF-β, Bcl-2, ERK, and its small splice variant LEDGF/p52 [52, 74–76]. A crosstalk between LEDGF/p75 activation and the STAT3/IL6 inflammatory pathway, implicated in PCa, has also been identified in HaCaT skin cancer cells and in breast cancer cells [41, 77]. Future studies will explore whether acquisition of taxane-resistance in mCRPC involves activation or suppression of LEDGF/p75 regulatory mechanisms.

## MATERIALS AND METHODS

### Cell lines, antibodies and reagents

The metastatic PCa cell lines DU145 (brain metastasis) and PC3 (bone metastasis) were purchased from the American Type Culture Collection (ATCC) (Cat.# HTB-81 and Cat.# CRL-1435, respectively). Cells were cultured following supplier's instructions in RPMI medium (Life Technologies – Thermo Fisher Scientific) supplemented with 10% fetal bovine serum (FBS), penicillin/streptomycin, and gentamicin. Cells were maintained in a humidified incubator with 5% CO2 at 37°C. The DTX-resistant cell line variants were developed as described previously [20]. Briefly, DU145 and PC3 cells were cultured in media containing 1 nM DTX and then surviving cells were passaged four times before increasing the concentration of DTX. This was repeated until resistant cells could be maintained with minimal cell death in media containing 10 nM DTX.

The following commercially acquired antibodies were used: rabbit polyclonal anti-LEDGF/p75 (1:1000, Bethyl Laboratories Inc. catalog# A300-848A); rabbit monoclonal anti-β-actin (1:5000, Cell Signaling catalog # 5125); rabbit polyclonal anti-cleaved caspase-3 (1:1000, Cell Signaling catalog # 9661); rabbit monoclonal anti-P-gp/MDR1/ABCB1 (1:1000, Cell Signaling catalog # 13342) and horseradish peroxidase (HRP)-labeled secondary IgG antibodies (goat anti-rabbit IgG, 1:5000, Thermo Fisher Scientific catalog # 31466; goat anti-human IgG/IgA/IgM, 1:5000, Thermo Fisher Scientific catalog # A18847). The human autoantibody to LEDGF/p75 (1:200) was from the serum bank of the Casiano Laboratory at the Center for Health Disparities and Molecular Medicine at Loma Linda University School of Medicine. The following cytotoxic drugs were used: DTX (LC-Laboratories), PTX (Sigma-Aldrich), and CBZ (Sanofi-Aventis). TNF-related apoptosis inducing ligand (TRAIL) was purchased from Peprotech and Actinomycin D was purchased from R&D Systems.

### Viability assays

PCa cells were treated with the different taxane drugs at the selected concentrations for up to 72 hr, or with TRAIL plus 100 ng/ml Actinomycin D for up to 24 hr. Cell morphology was visualized on an Olympus IX70 microscope equipped with Hoffmann Modulation Contrast (Olympus American) and a digital Spot Imaging System (Diagnostic Instruments). To assess viability, cells were seeded in 96-well plates at a density of 1×10^4^ cells per well and then treated with each individual drug in at least triplicates. A modified 3-(4,5-dimethylthiazol-2-yl)-2,5- diphenyltetrazolium bromide (MTT) assay (Sigma-Aldrich) was performed as described previously [18]. Absorbance was measured at 450 nm using a μQuant microplate reader (Bio-tek Instruments). Values were normalized to the absorbance obtained for the untreated, control cells. The approximate drug half-maximal effective concentration (EC50) was determined using the plotted values of the dose response curve. Each value represents the mean value of at least three different experiments in triplicates. The standard error of the mean (SEM) was calculated for each value.

### Quantitative real-time PCR

Quantitative Real-Time PCR (qPCR) was performed as described previously [28]. Briefly, Total RNA was extracted from cells using the RNeasy plus mini kit (Qiagen). The iScript cDNA synthesis kit (BioRad) was used to reverse transcribe RNA (0.5 μg) into cDNA. qPCR was performed using the MyiQ real-time PCR detection system with primers using iQ SYBR Green Supermix (BioRad) following manufacturers’ recommendations. Primer sequences for LEDGF/p75 were designed using the Primer3 software. Forward sequence (5′ to 3′) was TGCTTTTCCAGACATGGTTGT and reverse sequence (3′ to 5′) was CCCACAAACAGTGAAAAGACAG. Primers were commercially synthetized by Integrated DNA Technologies (IDT). Glyceraldehyde 3-phosphate dehydrogenase (GAPDH) mRNA was used for normalization. Data was normalized to values of corresponding controls and analyzed in three different experiments, each in triplicates.

### Immunoblotting procedures

Immunoblotting was performed as described previously [28]. Briefly, equal amounts of protein from whole cell lysates were separated using sodium dodecyl sulfate polyacrylamide gel electrophoresis (SDS-PAGE, NuPAGE 4–12%, Thermo Fisher Scientific) and transferred into polyvinyl difluoride (PVDF) membranes (Millipore). Membranes were blocked in 5% dry milk or 5% Bovine Serum Albumin (BSA), depending on the primary antibody, prepared in TBS-T buffer (20 mM Tris- HCl, pH 7.6, 140 mM NaCl, 0.1% Tween 20). Membranes were then probed individually with primary antibodies and corresponding secondary antibodies and washed several times with TBS-T between each antibody application. Enhanced chemiluminescence (ECL) was used to detect immunoreactive protein bands. For this, the ECL Western Blotting Substrate (Thermo Fisher Scientific Pierce, catalog # 32106) was added to the antibody-protein surface of each membrane, followed by incubation for 5 minutes. Membranes were then transferred to autoradiography cassettes and exposed to autoradiography films for different lengths of time to ensure accurate detection of immunoreactive protein bands.

### Indirect immunofluorescence microscopy

To visualize endogenous LEDGF/p75 expression, cells were grown on coverslips, washed with PBS, then fixed with 4% formaldehyde and permeabilized with 0.5% Triton X-100. Cells were then incubated with human anti-LEDGF/p75 serum autoantibody [47] at 1:200 dilution for one hour in a humid chamber. After washes with PBS, cells were incubated with a FITC-conjugated goat anti-human IgG (H+L) secondary antibody (1:200, Thermo Fisher Scientific catalog # 62-7111), used at 1:200 dilution. Cells were mounted and counterstained with medium containing 4′,6-diamidino-2- phenylindole (DAPI) (Vectashield). Images were acquired in a BioRevo Keyence BZ-9000 fluorescent microscope (Keyence). All images corresponding to a particular fluorescent dye were obtained using the same parameters.

### RNA interference-mediated knockdown of LEDGF/p75 in PCa cells

To achieve transient knockdown of LEDGF/p75 in our cellular models, specific short inhibitory RNAs (siRNAs) were used as described previously [20, 33]. Specifically, the si-LEDGF/p75 sequence corresponded to nucleotide sequence 1340–1360 (‘5-AGACAGCAUGAGGAAGCGAdTdT-3′), present in a region in the C-terminus of LEDGF/p75 that is not shared by its short alternative splice variant LEDGF/p52. Cells were transfected with 100 nM siRNAs using Oligofectamine (Life Technologies) following manufacturer's instruction. A scrambled siRNA duplex (siSD, Darmacon) was used as a negative control.

### Clonogenic assays

Cells with and without LEDGF/p75 knockdown were cultured at a density of 1×10^3^ per well in 6-well plates and treated with increasing concentrations of DTX or CBZ, or a single concentration of TRAIL combined with Actinomycin D. Cells were grown for 12 days, which is when the surviving colonies were visible. After removing the media, the colonies were washed with PBS and fixed using a 3:1 methanol:acetic acid solution. PBS was used to wash the remaining fixing solution and then a solution containing 0.5% crystal violet diluted in methanol was then added to stain the colonies. Finally, the crystal violet was removed by washing with water and plates were air dried overnight. Images of each individual plate were acquired using the VisionWorks Acquisition and Analysis software in a GelDoc-It^2^ imager (UVP, Analytik Jena Company). The parameters to obtain the images were the same for all the plates. The Automated Colony Counting capability of the software was used to count each colony in the individual wells using the same parameters for each plate. At least three plates from three independent experiments were used for quantification of colonies under a particular experimental condition or treatment.

### Statistical analysis

The Student's t-test (paired, two-tailed) was used to evaluate differences between treatment and control groups using Microsoft Excel. Differences were considered statistically significant at P values equal or below 0.05.

## Abbreviations

PCa: prostate cancer
DTX: docetaxel
PTX: paclitaxel
CBZ: cabazitaxel
TRAIL: TNF related apoptosis inducing ligand
